# National AED registries and coordinated AED systems as a system-level intervention to improve outcomes after out-of-hospital cardiac arrest: lessons from Japan for Poland

**DOI:** 10.3389/fpubh.2026.1823265

**Published:** 2026-04-15

**Authors:** Przemysław Żuratyński, Mai Taki Haratake

**Affiliations:** 1 Department of Emergency Medical Services, Faculty of Health Sciences, Nicolaus Copernicus University in Torun, Collegium Medicum in Bydgoszcz, Bydgoszcz, Poland; 2 Department of Nursing, Faculty of Nursing, Nishikyushu University, Ogi, Saga, Japan

**Keywords:** automated external defibrillator, public access defibrillation, out-of-hospital cardiac arrest, AED registry, emergency medical services, Japan, Poland

## Abstract

**Background:**

Out-of-hospital cardiac arrest (OHCA) survival depends critically on early defibrillation. Coordinated automated external defibrillator (AED) systems, including registries, mapping initiatives, and integration with emergency medical services (EMS), enable real-time device location, strategic placement, and system-level quality improvement. However, implementation varies widely between countries.

**Objective:**

To synthesize evidence on the impact of coordinated AED systems, including registries and public-access defibrillation programs, on OHCA outcomes and to identify lessons from Japan applicable to Poland.

**Methods:**

A systematic review was conducted according to PRISMA guidelines. PubMed/MEDLINE, EMBASE, Cochrane Library, and Scopus were searched (January 2015–June 2025) for studies evaluating AED registries, mapping systems, and public-access defibrillation programs. Eligible designs included randomized trials, observational registry studies, economic evaluations, and policy analyses. Inclusion criteria comprised studies evaluating AED registries, mapping systems, or public-access defibrillation in OHCA settings with reported clinical, operational, or economic outcomes. Exclusion criteria included studies without primary data, conference abstracts without full text, animal studies, and studies focused solely on in-hospital cardiac arrest. Study quality was assessed using the Newcastle–Ottawa Scale, AMSTAR 2, and CHEERS 2022.

**Results:**

Seventeen studies met inclusion criteria. Japan’s coordinated system, combining nationwide OHCA surveillance, public-access defibrillation programs, AED mapping initiatives, and EMS integration, has been associated in observational studies with increased bystander AED use among patients with bystander-witnessed OHCA with shockable rhythm (from 1.1 to 16.5%), reduced time to defibrillation, and improved neurological outcomes. In contrast, Poland currently lacks a fully coordinated national system, resulting in fragmented AED data and limited integration with emergency response. Modeling studies suggest that implementing a national system incorporating an AED registry, dispatcher integration, and community responder networks would be cost-effective.

**Conclusion:**

Coordinated AED systems, rather than standalone registries, represent an effective system-level approach to improving OHCA outcomes. Japan’s experience highlights the importance of integrating AED mapping, OHCA surveillance, EMS systems, and public training. Implementing such a coordinated approach in Poland could substantially improve survival and neurological outcomes after cardiac arrest. However, the observed benefits are likely multifactorial and reflect the combined effect of system-level interventions rather than a single component.

## Background

Out-of-hospital cardiac arrest (OHCA) remains one of the most pressing public health challenges globally, with survival outcomes strongly dependent on the speed and quality of the initial response. Among the key factors influencing survival are early recognition, prompt activation of emergency medical services (EMS), and, critically, early defibrillation, often achieved through the use of automated external defibrillators (AEDs) by bystanders (1–4). In recent years, national AED registries and coordinated AED mapping systems, including centralized or semi-centralized databases, that record the location, accessibility, and usage of AED devices, have emerged as essential components of advanced emergency response systems. These registries not only facilitate the strategic deployment of AEDs in high-incidence locations but also support real-time integration with dispatch centers, enabling rapid guidance for bystanders during cardiac emergencies.

Japan provides a notable example of the potential impact of such an approach. Through the implementation of a coordinated system combining public-access defibrillation programs, OHCA surveillance, and AED mapping initiatives, the country has achieved significant public health gains. Evidence demonstrates marked increases in bystander AED usage, substantial reductions in time-to-defibrillation, and higher rates of survival with favorable neurological outcomes among OHCA patients (2, 3, 5–9).

This success is likely attributable to the coordinated implementation of multiple system-level components, including public training, PAD programs, OHCA surveillance, and integration with emergency dispatch systems (2, 3). Importantly, Japan does not operate a single mandatory, centrally administered national AED registry. Instead, available evidence indicates that AED-related data and system performance are derived from a combination of nationwide OHCA registries and complementary research and implementation initiatives (2, 3, 10).

To better contextualize this structure, the Japanese system can be understood as comprising several distinct but complementary components. First, nationwide OHCA surveillance is conducted through Utstein-style registries based on data collected by emergency medical services and coordinated at the national level through the Fire and Disaster Management Agency (FDMA) (2, 3). Second, additional multicenter academic OHCA registries and registry-based analyses, including those coordinated by academic societies, provide more detailed clinical and epidemiological insights (10). Third, AED mapping and registration initiatives exist but are not organized within a single unified national framework and are instead developed through local or non-governmental efforts (10).

Although these components are not integrated within a single mandatory registry, they appear to operate in a complementary and partially coordinated manner, collectively supporting public-access defibrillation and contributing to improvements in system-level emergency response (2, 3, 10).

In contrast, Poland currently operates without a coordinated national system, including a centralized AED registry, resulting in fragmented data on AED availability, limited capacity to guide bystanders to the nearest device, and reduced ability to monitor and evaluate AED usage patterns and clinical outcomes (3, 11, 12). This gap restricts the optimization of AED placement and hinders evidence-based improvements to the national emergency response framework. Emerging research and modeling analyses indicate that introducing a nationwide AED registry in Poland, particularly when paired with large-scale community training initiatives and enhanced EMS integration, could be highly cost-effective. Such a system has the potential to meaningfully shorten response times, increase bystander intervention rates, and significantly improve survival and neurological outcomes for OHCA patients (3, 13, 14).

### Objective

This review synthesizes the evidence on the impact of coordinated AED systems, including registries, mapping initiatives, and public-access defibrillation programs, on out-of-hospital cardiac arrest outcomes, and identifies lessons from Japan applicable to improving emergency response systems in Poland.

## Methods

### Protocol

**Figure 1:**
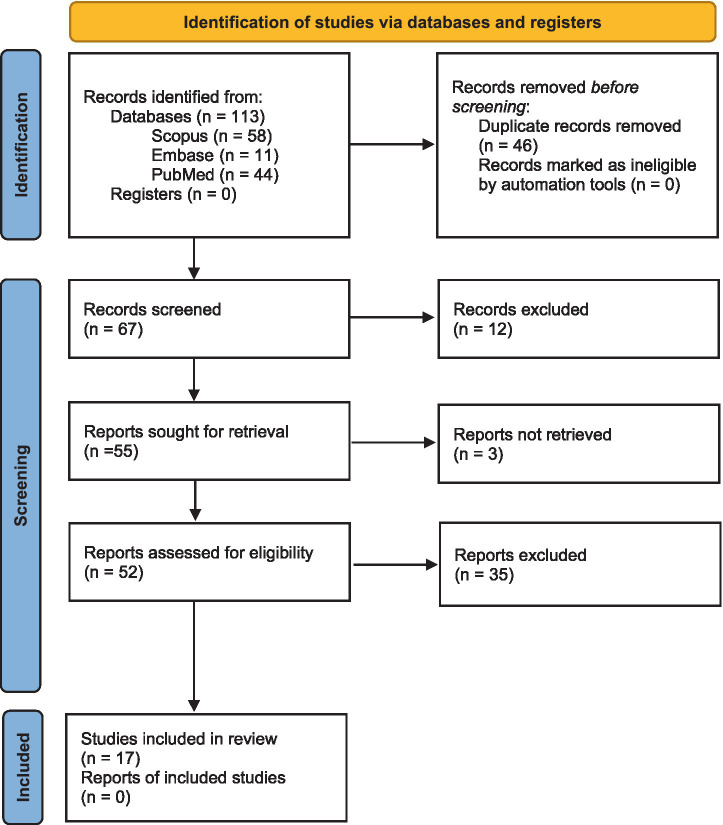
PRISMA.

This systematic review was conducted in accordance with the Preferred Reporting Items for Systematic Reviews and Meta-Analyses (PRISMA) guidelines (Figure 1).

### Search strategy

A comprehensive literature search was conducted in PubMed/MEDLINE, EMBASE, Cochrane Library, and Scopus from 01.01.2015 to 30.06.2025. Seminal studies published before 2015 were also included to provide essential background evidence. The search combined keywords and controlled vocabulary terms (MeSH, Emtree) relating to:

- “Automated External Defibrillator” OR “AED” OR “Public Access Defibrillation”
- “Registry” OR “Database” OR “Mapping” OR “Legislation” OR “Integration”
- “Emergency Medical Services” OR “EMS” OR “Dispatcher” OR “First Responder”
- “Out-of-Hospital Cardiac Arrest” OR “OHCA”
- “Japan” OR “Poland” OR “Europe” (for comparative context)

Boolean operators (“AND”/“OR”) were applied, and language filters were set to include studies published in English or Polish. Reference lists of eligible studies and relevant reviews were hand-searched to identify additional records.

### Eligibility criteria

Inclusion criteria:

- Population: Patients experiencing OHCA, EMS systems, or community-level AED programs.
- Intervention/Exposure: Implementation or evaluation of coordinated AED systems, including AED registries, mapping systems, or public-access defibrillation programs.
- Comparator: Settings without a registry, with partial coverage, or before registry implementation.
- Outcomes: AED use rates, time to defibrillation, survival to discharge, favorable neurological outcome, cost-effectiveness, or policy/operational impacts.
- Study design: Randomized controlled trials (RCTs), observational cohort studies, registry-based studies, health economics modeling, and comparative policy analyses.

Exclusion criteria:

- Studies without primary or registry-based data (e.g., opinion pieces, editorials).
- Conference abstracts without full-text data.
- Animal studies or in-hospital cardiac arrest only.

### Study selection

Two reviewers independently screened titles and abstracts for eligibility. Full texts of potentially relevant studies were retrieved and assessed against inclusion criteria. Disagreements were resolved through discussion or consultation with a third reviewer (Dr. Daniel Ślęzak), who acted as an independent adjudicator.

### Data extraction

Data were extracted independently by two reviewers using a standardized form, including:

- Study characteristics (authors, year, country, design, sample size).
- Type of AED system (registry, mapping system, PAD program, level of EMS integration).
- Main outcomes (AED use, time to defibrillation, survival, neurological outcome).
- Contextual information (legal framework, public training, accessibility).
- Methodological quality and risk of bias.

Characteristics and key findings of the included studies are summarized in Table 1.

**Table 1:** Study characteristics and key findings.

Study	Country	Year	Design	Sample size (*n*)	Key findings
Kitamura et al. (2)	Japan	2016	Nationwide cohort (Utstein registry)	43,762 OHCA cases (bystander-witnessed ventricular fibrillation)	Bystander AED use increased from 1.1 to 16.5%; PAD was associated with significantly higher 1-month favorable neurological survival (38.5% vs. 18.2%; adjusted OR 1.99), with attributable survivors increasing from 6 to 201 cases.
Kiyohara et al. (5)	Japan (Osaka)	2016	Observational registry study	9,978 OHCA cases	AED pads were applied in 3.5% of cases; among these, 29.6% received shocks. AED use was associated with significantly higher favorable neurological survival (19.4% vs. 3.0%; OR 2.76), despite low overall utilization.
Kiyohara et al. (6)	Japan	2019	Nationwide registry-based cohort	8,126 OHCA cases (shockable rhythm, bystander-witnessed)	28% received public-access defibrillation; among these, 58% were treated within 5 min, achieving 62% favorable neurological survival. Survival declined sharply with delays (to 7% at 21–25 min), with no survivors beyond 26 min.
Kobayashi et al. (8)	Japan	2019	Nationwide cohort	20,970 OHCA cases (bystander-witnessed, public locations)	PAD use varied widely by location (5.8–36.1%); PAD (AOR 2.33) and bystander CPR (AOR 1.78) were independently associated with improved neurological outcomes.
Żuratyński et al. (11)	Poland	2022	Retrospective observational	100 OHCA cases with AED use	AED was used in 100 public OHCA cases, with defibrillation performed in ~70%; however, lack of legal framework, incomplete EMS documentation, and limited data availability prevent reliable assessment of AED effectiveness.
Ślęzak et al. (12)	Poland	2022	Observational/descriptive	1,165 AED locations; 120 recorded uses	AED use remained low despite increasing availability; only 120 uses recorded across 1,165 locations, with temporal variability and increasing trend over time, reflecting limited system coordination and data integration.
Jaskuła et al. (13)	Poland	2025	Economic evaluation	—	CFR system was cost-effective (ICER €16,205/QALY), improving survival and neurological outcomes; all scenarios remained below willingness-to-pay threshold (€50,197/QALY).
Zadlo et al. (14)	Poland	2025	Registry-based cohort	8,253 OHCA cases	Bystander CPR (with ventilation) and AED use significantly increased ROSC; dispatcher-assisted CPR improved ROSC (22.1–30.6%), highlighting the critical role of early intervention despite system limitations.
Baldi et al. (15)	Europe	2021	Multinational observational	28 countries; ~28,000 OHCA cases (≈17,000–18,000 analyzed in Utstein comparator group)	Substantial variability across Europe; AED use before EMS ranges from 0 to >50%, with generally low bystander use (<10%) and wide variation in ROSC and survival (≈5–50%).
Moens et al. (16)	Belgium and Switzerland FR systems	2023	Comparative implementation study	Not applicable (system-level analysis, 2006–2022)	Structured 4-step roadmap for FR systems; integration of AED mapping and responder networks improves early response and survival.
Fredman et al. (17)	Sweden	2018	Nationwide registry-based observational study	6,703 AEDs (validated); 7,241 registered AEDs in workplaces	Number of AEDs doubled between 2013 and 2016; however, a substantial proportion remained unregistered (only ~57% registered in one region), with lack of awareness (74.5%) as the main barrier.
O’Sullivan et al. (18)	UK	2024	National implementation study	82,108 AEDs registered	Centralized national AED network implemented across all 14 ambulance services; 54% of AEDs were newly identified, and total registered devices doubled, improving real-time EMS access to AED data.
Mao and Ong (19)	—	2016	Narrative review	Not applicable	AED accessibility and early defibrillation are key determinants of survival; improving public access and strategic AED placement increases bystander defibrillation and outcomes
Jonsson et al. (20)	—	2023	Narrative review	Not applicable	Integration of AEDs with first responder systems improves early defibrillation rates and survival; rapid activation of nearby responders is critical for effective AED use.
Elrod et al. (21)	USA	2017	Registry protocol	Not applicable	Dynamic AED registry designed to support surveillance, integration, and evaluation of AED systems.
Karlsson et al. (22)	Denmark	2019	Registry-based cohort	2,500 OHCA cases	AED availability significantly increased bystander defibrillation (~3-fold: 13.8% vs. 4.8%) and nearly doubled 30-day survival (72.7% vs. 44.1%); however, <50% of AEDs were accessible at the time of OHCA.
Hansen et al. (23)	Denmark	2017	Registry-based cohort	18,688 OHCA cases	Bystander defibrillation increased markedly in public locations (1.2–15.3%), but remained unchanged in residential settings (~1.3%); 30-day survival after bystander defibrillation rose to 57.5% in public vs. 25.6% in residential locations.

### Quality assessment of included studies

The methodological quality of included studies was independently assessed by two reviewers using tools appropriate to study design:

- Newcastle–Ottawa Scale (NOS) for observational studies,
- AMSTAR 2 for systematic reviews,
- CHEERS 2022 for economic evaluations.

Studies scoring ≥7 points on the NOS were classified as high quality, 5–6 points as moderate quality, and ≤4 points as low quality. Disagreements in quality assessment were resolved by consensus. Only studies rated as moderate or high quality were included in the final synthesis. A detailed study-level quality assessment table is provided (Table 2).

**Table 2:** Methodological quality assessment of included studies.

Study	Tool used	Quality rating	Risk of bias
Kitamura et al. (2)	NOS	High (8/9)	Low
Kiyohara et al. (5)	NOS	High (7/9)	Low
Kiyohara et al. (6)	NOS	High (8/9)	Low
Kobayashi et al. (8)	NOS	High (7/9)	Low
Żuratyński et al. (11)	NOS	Moderate (5/9)	Moderate
Ślęzak et al. (12)	NOS	Moderate (5/9)	Moderate
Jaskuła et al. (13)	CHEERS 2022	High (CHEERS compliant)	Low
Zadlo et al. (14)	NOS	Moderate (6/9)	Moderate
Baldi et al. (15)	NOS	Moderate (5/9)	Moderate
Moens et al. (16)	NOS	Moderate (5/9)	Moderate
Fredman et al. (17)	NOS	High (7/9)	Low
O’Sullivan et al. (18)	NOS	Moderate (5/9)	Moderate
Mao and Ong (19)	AMSTAR 2	Moderate	Moderate
Jonsson et al. (20)	AMSTAR 2	Moderate	Moderate
Elrod et al. (21)	—	Not applicable (study protocol)	—
Karlsson et al. (22)	NOS	High (8/9)	Low
Hansen et al. (23)	NOS	High (8/9)	Low

### Data synthesis

Due to heterogeneity in study designs, populations, and outcome measures, a narrative synthesis approach was used. Findings were grouped according to system components (e.g., AED registries, mapping systems, PAD programs) and outcomes.

## Results

### Attributes and implementation of coordinated AED systems

Japan has developed a coordinated system combining nationwide OHCA surveillance based on Utstein-style registries, public-access defibrillation (PAD) programs, and AED deployment strategies supported by governmental, academic, and local initiatives (2, 5, 6, 8).

Nationwide analyses demonstrated a substantial increase in bystander AED use in shockable OHCA cases. Among 43,762 bystander-witnessed ventricular fibrillation cases, 10.3% (4,499 patients) received public-access defibrillation. The proportion of patients receiving PAD increased from 1.1% in 2005 to 16.5% in 2013 (*p* < 0.001 for trend) (2).

Public-access defibrillation was associated with improved outcomes. One-month survival with favorable neurological status was higher in patients treated with PAD compared to those without defibrillation (38.5% vs. 18.2%; adjusted odds ratio 1.99; 95% CI: 1.80–2.19) (2). The number of patients achieving favorable neurological survival attributable to PAD increased from 6 cases in 2005 to 201 cases in 2013 (*p* < 0.001 for trend) (2).

In Osaka, AED pads were applied in 3.5% of OHCA cases (351/9,978), and favorable neurological outcomes were higher among patients receiving AED intervention compared to those without (19.4% vs. 3.0%; OR 2.76; 95% CI: 1.92–3.97) (5).

Among 8,126 bystander-witnessed OHCA cases with shockable rhythm, 28% (2,282/8,126) received public-access defibrillation. When defibrillation was performed within 5 min of collapse, 62% of patients achieved favorable neurological outcomes at 1 month. This probability decreased with increasing time to defibrillation (48% for 6–10 min, 38% for 11–15 min, 30% for 16–20 min, and 7% for 21–25 min; *p* for trend <0.001), with no survivors beyond 26 min (6).

In a nationwide cohort of 20,970 bystander-witnessed OHCA cases in public locations, PAD use ranged from 5.8% on streets and highways to 36.1% in educational institutions. Both bystander CPR (AOR 1.78; 95% CI: 1.54–2.07) and PAD (AOR 2.33; 95% CI: 2.05–2.66) were associated with favorable neurological outcomes (8).

In Poland, AED-related data are limited and fragmented. A retrospective analysis of public-space OHCA cases between 2015 and 2020 identified 100 instances of AED use, with defibrillation performed in approximately 70% of cases (11). Analyses of AED deployment identified approximately 1,165 publicly accessible AED locations and 120 recorded uses between 2008 and 2018 (12).

### AED use and OHCA outcomes in Japan

Japan’s coordinated system, combining nationwide OHCA surveillance, public-access defibrillation programs, AED mapping initiatives, and EMS integration, has yielded measurable and substantial improvements in cardiac arrest outcomes. Over less than a decade, the rate of bystander AED use in shockable OHCA cases increased more than fifteen-fold, from 1.1% (95% CI: 1.0–1.2) in 2005 to 16.5% (95% CI: 16.0–17.0) in 2013 (2, 5–8). This surge in early intervention by lay rescuers translated directly into better patient prognoses. Nationwide data show that the proportion of patients achieving favorable neurological outcomes 1 month after cardiac arrest more than doubled when a public-access AED was used 38.5% (95% CI: 36.9–40.1) compared with 18.2% (95% CI: 17.5–18.9) in cases without such intervention (2, 5, 6, 8). These improvements are likely multifactorial and cannot be attributed to a single component, such as an AED registry alone, but rather to the combined effect of coordinated system-level interventions.

One of the key mechanisms behind this improvement is the marked reduction in time to defibrillation. Registry analyses revealed a decrease in the mean time from collapse to shock delivery, from 3.7 min (95% CI: 3.6–3.8) to 2.2 min (95% CI: 2.1–2.3) over the same period (3). This time gain is clinically critical, as every minute of delay in defibrillation reduces the likelihood of survival with good neurological function by approximately 7–10% (adjusted HR for each 1-min delay = 0.91; 95% CI: 0.88–0.94) (3, 6, 8). Importantly, early defibrillation was consistently associated with improved outcomes regardless of who delivered the shock—whether a trained medical professional or a lay bystander (adjusted OR for favorable neurological recovery with early defibrillation = 2.12; 95% CI: 1.95–2.31) (6, 8).

### Lessons for Poland: current status and potential benefits

In Poland, the use of automated external defibrillators in public spaces has been steadily increasing in recent years, reflecting growing public awareness and gradual expansion of device availability. However, the lack of a coordinated national system, including a centralized AED registry significantly limits the ability to systematically monitor device locations, usage patterns, and clinical outcomes (11, 12). Without centralized, standardized data, policymakers and emergency services cannot fully assess coverage gaps, identify high-priority placement areas, or evaluate the effectiveness of existing deployments.

Available evidence from Poland highlights the scale of current system limitations. For example, regional analyses indicate that despite increasing AED availability, actual bystander AED use remains very low, with reported rates below 5% in some regions. Furthermore, existing AED databases are typically based on voluntary reporting and are not regularly updated, limiting their reliability for real-time emergency use. These findings confirm that the absence of a centralized and mandatory registry significantly constrains the effectiveness of public-access defibrillation programs at the national level (11, 12).

Research consistently emphasizes that establishing a legal framework mandating AED registration is a critical first step toward optimizing public-access defibrillation in Poland (11, 12). Mandatory reporting, combined with regular updates on AED location and accessibility, would create a reliable national database that could be directly linked to emergency dispatch systems. Such integration would allow dispatchers to guide callers or bystanders to the nearest available AED in real time, thereby shortening the time to defibrillation, a key factor in improving survival after out-of-hospital cardiac arrest (OHCA).

Modeling and health economics studies further suggest that implementing a coordinated national system, comprising a comprehensive AED registry, widespread community training in basic life support, and real-time integration with emergency dispatch, would not only be cost-effective but also lead to substantial public health gains (13, 14). Projections indicate that such measures could significantly increase bystander AED use, improve survival rates, and enhance the proportion of patients achieving favorable neurological outcomes. This evidence supports the urgent need for a national strategy to standardize AED data collection, improve accessibility, and embed AED use as an integral part of Poland’s emergency response framework.

It should also be noted that several local and regional AED mapping initiatives exist in Poland, including municipal databases and volunteer-based online platforms. However, these systems are fragmented, incomplete, and lack standardization, which significantly limits their utility for emergency dispatch and national planning. Their existence nevertheless demonstrates both the feasibility of AED mapping and the need for integration into a unified national registry.

### International comparisons and barriers

Across Europe, research demonstrates substantial heterogeneity in how countries regulate, map, and maintain registry systems for automated external defibrillators. Legislative frameworks vary widely, from nations with mandatory AED registration, regular data reporting, and legal requirements for AED placement in public spaces, to those with entirely voluntary systems and no standardized national oversight (15–18). This variation is mirrored in operational practices: some countries maintain dynamic, real-time AED mapping integrated with emergency dispatch and first responder alert networks, while others rely on static, incomplete, or outdated lists that limit their utility during emergencies.

Evidence consistently shows that countries with well-developed AED systems, including registries, mapping tools, and coordinated first responder networks achieve significantly higher rates of bystander AED use and improved survival after out-of-hospital cardiac arrest (15–18). In these settings, registry data supports not only operational response, by directing rescuers to the nearest device, but also strategic planning for AED placement, monitoring of usage patterns, and public health research.

Despite these clear benefits, the implementation of national AED registries remains challenging in many European countries. Barriers include complex legal considerations, such as data protection and liability issues. Logistical difficulties in maintaining accurate, up-to-date device records; and organizational fragmentation, particularly where responsibilities are split between multiple agencies (11, 15, 17). Additionally, limited public awareness of AED availability, insufficient training in basic life support, and low confidence among lay rescuers further reduce the potential impact of AED programs. Overcoming these barriers requires coordinated policy action, sustainable funding models, and large-scale community engagement to ensure that registries are accurate, accessible, and effectively integrated into the emergency response chain.

### Cost-effectiveness and policy implications

Modeling studies conducted in Poland indicate that the implementation of a nationwide community first responder (CFR) system, fully integrated with automated external defibrillators and supported by a coordinated national system incorporating an AED registry, is expected to be cost-effective based on modeling studies. Such a system would enable rapid mobilization of trained volunteers in proximity to an out-of-hospital cardiac arrest and provide them with real-time guidance to the nearest available AED. Simulation models show that this approach could substantially improve survival rates and increase quality-adjusted life years (QALYs) gained, while keeping costs within thresholds typically considered acceptable for public health interventions in Poland (13, 14). The cost-effectiveness is driven by earlier defibrillation, higher rates of bystander intervention, and improved neurological outcomes among survivors, which in turn reduce the long-term healthcare and social costs associated with severe post-arrest disability.

The available economic evaluations were primarily conducted from the healthcare system perspective and incorporated key parameters such as response time reduction, increased bystander intervention rates, and improvements in survival with favorable neurological outcomes. Most models applied a long-term time horizon and used quality-adjusted life years (QALYs) as the primary outcome measure. Sensitivity analyses consistently confirmed the robustness of the findings across a range of assumptions.

International evidence reinforces these findings, demonstrating that jurisdictions with strong CFR networks, mandatory AED registration, and full integration of AED location data into emergency medical services (EMS) dispatch achieve markedly better OHCA outcomes (19–21). In these systems, dispatchers can instantly direct first responders and bystanders to the closest AED, minimizing time to shock delivery, a factor consistently linked to survival and good neurological recovery. Moreover, pairing registry implementation with widespread public training in basic life support (BLS) ensures that AED accessibility translates into actual device use. These results support the development of national policies in Poland mandating AED registration, expanding community training programs, and embedding AED data integration into EMS operations as essential steps toward improving cardiac arrest survival nationwide.

These assumptions are consistent with established health economic evaluation frameworks for public health interventions.

A structured summary of key findings and supporting evidence is presented in Table 3.

**Table 3:** Summary of evidence and supporting references.

Claim	Evidence strength	Reasoning	Papers
National AED registries integrated with EMS improve OHCA survival and neurological outcomes	Strong	Multiple large-scale, population-based studies in Japan and Denmark show doubled survival and favorable outcomes with registry-driven AED use	Kitamura et al. (2), Kiyohara et al. (6), Fredman et al. (17), and Karlsson et al. (22)
Public-access AED use increases with registry-linked dispatcher systems and public training	Strong	Registry and dispatcher integration tripled bystander defibrillation rates; public training further boosts AED use	Kitamura et al. (2), Kiyohara et al. (5), Ślęzak et al. (12), Baldi et al. (15), Fredman et al. (17), and Hansen et al. (23)
Lack of a national AED registry in Poland leads to fragmented data and low AED use	Moderate	Polish studies show only ~100 documented public AED uses in 5 years; no legal mandate for registration	Kobayashi et al. (8), Żuratyński et al. (11), and Ślęzak et al. (12)
Implementing a national registry and CFR system in Poland is cost-effective	Moderate	Modeling shows improved survival and QALYs at acceptable cost; supports policy adoption	Jaskuła et al. (13), and Zadlo et al. (14)
AED accessibility (24/7) is critical for maximizing impact	Moderate	Studies show >50% coverage loss due to limited AED access; 24/7 access nearly doubles survival	Kiyohara et al. (5), Mao and Ong (19), and Karlsson et al. (22)
Dispatcher referral to AEDs remains underutilized even with registry tools	Moderate	Swedish study found only 4.3% of eligible cases received dispatcher referral to AED	Fredman et al. (17), and O’Sullivan et al. (18)

## Discussion

The available evidence provides strong and consistent support for the critical role of coordinated AED systems, including registries, mapping initiatives, and public-access defibrillation programs, in enhancing emergency response systems and improving outcomes after out-of-hospital cardiac arrest. In this review, a “coordinated AED system” refers to a multi-component, system-level framework integrating AED registries or mapping systems, public-access defibrillation programs, OHCA surveillance, and EMS dispatch, supported by public training and appropriate legal infrastructure. The evidence from multiple countries indicates that these coordinated system-level strategies, when integrated with emergency medical services and supported by public training and appropriate legal frameworks, can significantly improve survival and neurological outcomes after OHCA (1, 2, 5, 6, 22–24). Japan’s experience offers a compelling example of how a coordinated system integrating PAD programs, nationwide OHCA registries, AED mapping initiatives, and EMS systems can produce substantial public health gains. Recent evidence further clarifies that Japan’s system is not based on a single unified AED registry, but rather on a multi-layered registry infrastructure combining nationwide OHCA surveillance, academic registries, and complementary initiatives, which together support system-level improvements in OHCA outcomes (10). Over the past two decades, Japan has demonstrated that such a system can dramatically increase bystander AED use, significantly shorten the time from collapse to defibrillation, and markedly improve both survival rates and the proportion of patients achieving favorable neurological outcomes (2, 3, 5–9). The study by Nakagawa et al. (25) analyzed nationwide Japanese data to identify factors linked with the provision of public access defibrillation (PAD) during OHCA events. Results showed that PAD was more likely when cardiac arrests occurred in public locations, were witnessed, and when bystanders were trained or alerted through dispatcher-assisted CPR. The authors concluded that enhancing public training, expanding AED coverage in high-traffic areas, and strengthening dispatcher guidance could significantly increase PAD rates and improve survival outcomes. The study by Ishii et al. (26) examined nationwide Japanese OHCA data to explore sex- and age-based disparities in public access defibrillation (PAD), bystander CPR, and neurological outcomes. Findings revealed that women and older adults were less likely to receive PAD and bystander CPR compared to men and younger patients, even when arrests were witnessed in public locations. These disparities contributed to lower rates of favorable neurological recovery. The authors emphasized the need for targeted public education and training campaigns to address unconscious biases and ensure equitable emergency response for all demographic groups. Importantly, these improvements should not be attributed to AED registries alone, but rather to the synergistic effect of multiple system components, including PAD programs, OHCA surveillance, EMS integration, and widespread public training (2, 3, 10). This evidence supports the interpretation of a coordinated, system-level approach rather than the effect of a single national AED registry, consistent with recent literature describing Japan’s multi-component registry ecosystem (10).

A defining feature of Japan’s model is the observed interaction between OHCA surveillance systems, AED availability, and EMS dispatch, although these components are not formally integrated within a single national system (10). This coordinated system should be understood as a conceptual synthesis of evidence from multiple sources rather than a formally defined or centrally governed national program. As highlighted in recent reviews, Japan’s registry landscape consists of multiple overlapping systems, including FDMA-based nationwide OHCA surveillance and academic registries, rather than a single centralized AED registry (10). This connection allows dispatchers to provide real-time directions to the nearest device, ensuring rapid initiation of defibrillation. At the same time, continuous, nationwide data collection on OHCA events, combined with complementary data on AED use and accessibility, enables ongoing system evaluation and optimization (3, 9, 10). This feedback loop between data and practice has been instrumental in sustaining long-term improvements. In Japan, between 2005 and 2020, a nationwide initiative was implemented to promote widespread civilian cardiopulmonary resuscitation training (27). During this period, the cumulative number of certified citizens increased from 9,930,327 in 2005 to 34,938,322 in 2020, covering 32.3% of the population aged 15 years and older (27–29). The annual growth of trained individuals showed a steady upward trend [incidence rate ratio (IRR) = 1.03; 95% CI: 1.03–1.03; *p* < 0.001] (27). At the same time, the prevalence of citizen-initiated CPR significantly increased. In 2005, bystander CPR was performed in 40.6% of out-of-hospital cardiac arrest cases, compared with 56.8% in 2020 (*p* for trend <0.001). Multivariable analyses confirmed that the rise in bystander CPR was an independent factor associated with improved survival after OHCA (27). Data from the Japanese OHCA registry further demonstrated that patients who received bystander CPR were more likely to survive to hospital discharge with favorable neurological outcomes compared to those without intervention. These findings are consistent with earlier studies from Japan, which showed that public-access defibrillation and widespread citizen CPR training significantly improve survival rates in shockable rhythms (2, 3, 5, 28–30). The Japanese experience highlights that sustained, nationwide educational policies—including mandatory CPR training in schools and broad accessibility of adult training courses—can effectively increase bystander intervention and improve survival outcomes. Comparable results have been reported in Sweden and Denmark, where comprehensive AED registries and community training programs were associated with higher rates of bystander defibrillation and improved outcomes (17, 22, 23). For countries such as Poland, where significant gaps remain in AED registration and widespread public training, adopting a similar strategy could represent a key step toward improving survival following OHCA (11–13, 31).

In contrast, Poland’s lack of a coordinated national system, including a centralized AED registry, represents a significant structural gap in its emergency care framework. Without a centralized, legally mandated system, the country struggles to coordinate AED deployment, monitor device usage, or systematically analyze outcomes. As a result, opportunities to improve OHCA survival through faster, more coordinated public-access defibrillation are frequently missed (3, 11, 12).

Both modeling and observational studies from Poland indicate that introducing a national AED registry, especially if integrated with emergency dispatch systems and paired with public training programs, would be a highly cost-effective intervention (13, 14). Simulations suggest such a system could substantially increase bystander intervention rates, reduce response times, and improve survival and neurological outcomes. However, several barriers must be addressed before such a system can be successfully implemented. These include establishing an enabling legal framework, raising public awareness of AED use, ensuring sustainable funding, and overcoming logistical and organizational challenges related to data maintenance and system integration (11, 15, 17).

Addressing this gap will require overcoming several well-documented barriers. These include limited accessibility of AEDs in public and residential areas, the lack of real-time dispatcher integration with AED location databases, and insufficient public awareness and confidence in AED use. Evidence from international models suggests that tackling these challenges, through legal mandates for AED registration, integration with EMS dispatch, and large-scale community training, could replicate the benefits observed in Japan, Denmark, and Sweden.

From a health economics perspective, the case for action in Poland is strong. Modeling studies demonstrate that implementing a national AED registry, combined with a community first responder system, would be highly cost-effective. Such an initiative is projected to yield substantial gains in survival and quality-adjusted life years at costs well within acceptable thresholds for public health interventions (11, 18). These findings provide a clear policy direction: invest in a coordinated, legally supported national AED registry integrated with EMS and supported by public training to maximize the life-saving potential of early defibrillation. The study by Zmushka et al. (32) found that in the hilly Nagasaki Medical Region, EMS response time and AED accessibility are critical determinants of OHCA survival. Analysis of regional Utstein registry data showed that early defibrillation and bystander CPR significantly improved outcomes, but delays were more common in difficult terrain. The findings highlight the need for strategic AED placement, 24/7 accessibility, and development of community first responder networks, recommendations relevant for rural areas in Poland as well. The study by Bujak et al. (31) found that in Upper Silesia, Poland, bystander AED use during OHCA was extremely low (4.6%), despite over half of cases receiving bystander CPR. The findings highlight a critical gap in AED accessibility and utilization, underscoring the need for broader public AED coverage and training.

The strength of the evidence varies considerably between countries. In Japan, large-scale, population-based registry studies supported by robust statistical analyses provide high-quality evidence derived from nationwide OHCA registries and coordinated system-level interventions rather than from a single national AED registry (2, 3, 10). In Poland, however, the current body of evidence is more limited, relying primarily on smaller cohort studies, local initiatives, and predictive modeling. This disparity underscores both the proven benefits of well-functioning, integrated AED systems supported by registry-based surveillance and coordinated system-level interventions and the urgent need for more comprehensive, real-world data from Poland to guide policy development and system design. The ENSURE study revealed substantial variation across Europe in AED legislation, mapping systems, and usage rates (15). Countries with well-developed AED systems, including registries, mapping tools, and coordinated first responder networks, achieve higher rates of bystander AED use and improved survival after OHCA. These findings underscore the critical role of robust legal frameworks and registry, EMS integration in enhancing the effectiveness of prehospital emergency response.

Importantly, the current lack of comprehensive national data in Poland underscores the need not only for implementation but also for prospective evaluation of registry-based interventions. Establishing a national AED registry would enable continuous system monitoring and provide high-quality real-world evidence to inform future policy decisions. This gap highlights a critical opportunity for Poland to transition from fragmented, reactive approaches to a data-driven, system-level strategy for OHCA management. Future research should focus on evaluating coordinated system-level interventions rather than isolated components, to better understand their relative contributions to improved OHCA outcomes.

## Conclusion

Coordinated AED systems, including registries, mapping initiatives, public-access defibrillation programs, and integration with emergency medical services, represent an effective and potentially cost-effective system-level approach to improving outcomes after out-of-hospital cardiac arrest. Japan’s experience demonstrates how the coordinated implementation of these components can substantially increase bystander AED use, shorten time to defibrillation, and improve survival and neurological outcomes.

Importantly, these benefits are likely multifactorial and cannot be attributed to a single component, such as an AED registry alone, but rather to the combined effect of integrated system-level interventions.

For Poland, the key implication is not only the implementation of a national AED registry, but the development of a coordinated national system incorporating registry infrastructure, EMS integration, widespread public training, and supportive legal frameworks. Such an approach could significantly increase survival rates and the proportion of patients achieving favorable neurological outcomes after cardiac arrest.

Despite strong international evidence, there remains a lack of real-world data in Poland on the implementation and outcomes of coordinated AED systems. Barriers such as limited AED accessibility, lack of integration with dispatcher systems, and low public awareness remain insufficiently explored. There is also a notable gap in data regarding AED use in residential settings, legal and organizational challenges, and the effectiveness of public education strategies.

Future research should focus on the implementation and evaluation of coordinated system-level interventions, including but not limited to AED registries, to better understand their impact on OHCA outcomes and inform evidence-based policy development.

## Data Availability

The original contributions presented in the study are included in the article/supplementary material, further inquiries can be directed to the corresponding author.
